# Short-Term Oral Sorafenib for Therapy of Intratumoral Shunts of Hepatocellular Carcinoma to Enable Intraarterial Treatment

**DOI:** 10.1007/s00270-019-02294-7

**Published:** 2019-07-30

**Authors:** J. Thüring, M. Zimmermann, P. Bruners, F. Pedersoli, M. Schulze-Hagen, E. Barzakova, C. K. Kuhl, P. Isfort

**Affiliations:** Department of Diagnostic and Interventional Radiology, Aachen University Hospital, Pauwelsstreet 30, 52072 Aachen, Germany

**Keywords:** HCC, Sorafenib, Shunt, Fistula

## Abstract

**Introduction:**

Significant intratumoral shunts between tumor-supplying arteries and portal or liver veins are a contraindication for transarterial therapy of HCC because interventional treatment of these shunts is frequently insufficient. Sorafenib has anti-angiogenic effects and is indicated for palliative treatment of patients with HCC. Here, we report our experience with the use of sorafenib for the closure of intratumoral shunts in patients scheduled for transarterial therapy of HCC.

**Materials and Methods:**

Three patients with HCC, aged 65, 82 and 79 years, exhibited a significant intratumoral shunting from tumor artery to portal (*n* = 1) or liver veins (*n* = 2). In all cases, intratumoral shunting had already been suspected based on pre-interventional CT angiography, and DSA confirmed the shunt. Oral sorafenib (800 mg/day) was administered for at least four weeks, only and specifically to occlude the shunt. Hereafter, patients were re-evaluated by CT and DSA.

**Results:**

All patients tolerated the full prescribed dose for at least 4 weeks. In one case, therapy was prolonged with an adapted dose (400 mg/day) due to sorafenib-related hand–foot syndrome. After sorafenib treatment, CT and DSA confirmed a complete closure of intratumoral shunts for all patients. No tumor progression was observed. All three patients hereafter underwent successful transarterial treatment by TACE (*n* = 2) or TARE (*n* = 1) without complications. Progression-free survival according to mRECIST was 501, 397 and 599 days, respectively.

**Conclusion:**

Even short-term oral sorafenib seems to effectively close intratumoral shunts in patients with HCC and thus might enable transarterial treatment of these patients.

**Electronic supplementary material:**

The online version of this article (10.1007/s00270-019-02294-7) contains supplementary material, which is available to authorized users.

## Introduction

Hepatocellular carcinoma (HCC) frequently presents with vascular invasion. In fewer cases, this vascular invasion includes the formation of intratumoral shunts (ITSs) between a liver artery and a portal or liver vein [1]. In contrast to micro-ITS—which only presents by an elevated hepatopulmonary shunt (HPS)—macro-ITS can be visualized on computed tomography (CT) or digital subtraction angiography (DSA) and can be a contraindication for transarterial therapies, i.e., transarterial chemoembolization (TACE) [2] or transarterial radioembolization (TARE) [3]. Ngan and Peh [4] reported an overall incidence for a significant ITS in HCC of 31.2% in a cohort of 292 patients; the majority showed an arterioportal shunt (28.8%), whereas arteriovenous shunting was less frequently observed (2.4%). To date, closure of a significant ITS was carried out with various interventional approaches. Murata et al. [5] showed that a temporary portal vein occlusion with a balloon catheter might be superior to an occlusion of the fistula with microcoils and absorbable gelatin sponge before TACE; however, these approaches could only provide short-term benefits [6, 7].

Sorafenib is an orally administered tyrosine kinase inhibitor, with anti-angiogenic effects, and is indicated as palliative therapy in advanced-stage HCC [8]. Since the initial approval of sorafenib in 2006, some case reports documented closure of shunts after sorafenib administration, thus indicating it as a potential therapy option [9, 10].

Out of this and based on our clinical experience with sorafenib, we aim to increase the clinical data basis on these rare cases of a significant otherwise untreatable ITS treated with oral sorafenib specifically with the intention to close the ITS and to hereafter perform TACE or TARE safely.

## Materials and Methods

### Clinical Observation

Between January 2014 and January 2019, three patients (one woman and two men; mean age 75.3 ± 12.3 years) with intermediate-stage HCC were excluded from transarterial therapy because of a significant ITS. In all cases, ITS was already seen on pre-interventional CT and confirmed by DSA. The multidisciplinary tumor board (MDT) indicated in these three cases oral sorafenib (Nexavar, Bayer Schering Pharma, Berlin, Germany) only and exclusively as an attempt to close the ITS. In every case, sorafenib administration takes at least 4 weeks with a daily dose of 800 mg (2 × 400 mg).

After the initial diagnosis of the significant ITS on CT and confirmation by DSA, all patients were discussed in the MDT within 1 week after diagnosis. Oral sorafenib administration was started with a perspective duration of 4 weeks. Patients were hereafter re-evaluated by CT, and if ITS was absent, they were scheduled for intraarterial treatment. If ITS was still present, oral sorafenib was prolonged again for 4 weeks.

CT angiography was performed with clinical CT systems (Somatom Definition Flash and Somatom Definition AS, Siemens Medical Systems, Forchheim, Germany). Arterial and portal venous phases were acquired following body-weight-adapted application of iodinated contrast media (1.5 ml per kilogram of body weight; iopromide 370 mg/ml, Ultravist, Bayer, Leverkusen Germany).

“Direct hepatic arteriography was carried out on a monoplanar DSA system (Artis zee/zeego, Siemens Healthcare, Erlangen, Germany).” A 5-F Cobra 2 catheter (Terumo, Leuven, Belgium) was placed in the common hepatic arteria for hepatography, followed by a tumor-selective angiography with a 2.5-F microcatheter (Renegade, Boston Scientific, USA) in the tumor-supplying artery. Contrast media were applicated using a power injector (4 ml/s and 1.5 ml/s, respectively).

Super-selective TACE was performed by cannulation of the tumor-supplying arteries with a microcatheter. A cone beam CT with contrast administration via the microcatheter was performed in order to confirm the correct position of the microcatheter. A mixture of iodinated contrast media 5 ml and an emulsion of doxorubicin 50 mg (Adriablastin, Pfizer, USA) with 10 ml lipiodol (Laboratoire Guerbet, Aulnay-sous-Bois, France) was injected until stasis was visualized. No antibiotic prophylaxis was administered.

TARE patients underwent preparative intervention with angiography of the hepatic artery and protective coiling of relevant side branches (e.g., gastroduodenal artery, right gastric artery). Afterward, 150 MBq ^99m^Tc-MAA was injected into the liver arteries using the same catheter position chosen for the scheduled TARE session. The hepatopulmonary shunt fraction and tracer distribution were evaluated with subsequent planar images and SPECT imaging. TARE was performed using Y^90^-loaded resin microspheres. The activity and dose for Y^90^-Spheres were calculated according to the body surface model as suggested by the REBOC expert panel [11]. SIRT was performed in a lobar approach.

## Results

### Clinical Observation

**Fig. 1:**
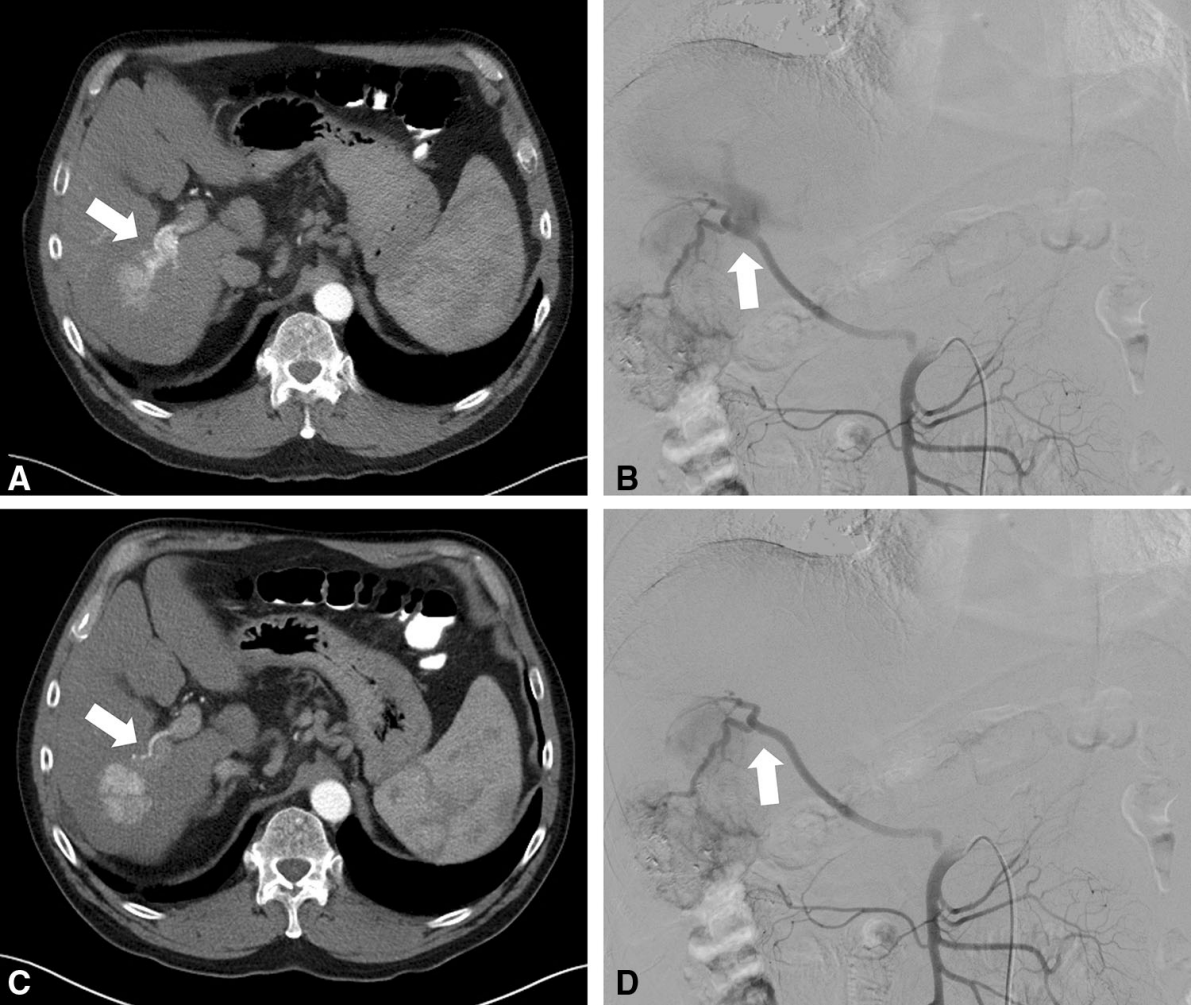
A 65-year-old patient with multifocal HCC based on ethyl toxic liver cirrhosis. **A** An arterioportal fistula (arrows) can be delineated based on the CT image. **B** DSA confirms the large arterioportal fistula (white arrow) with high-flow conditions. **C** After the treatment with sorafenib, the fistula cannot be visualized (white arrow). **D** Technical, uncomplicated and safe TACE could be carried out under occluded fistula (white arrow)

A 65-year-old man was assigned to our department with suspicious multifocal liver lesions due to ethyl toxic cirrhosis. CT showed multifocal HCCs with a solitary significant ITS in segment VI, and hence, early contrast enhancement of the right portal vein can be seen (Fig. 1A). Selective arteriography confirmed the ITS (Fig. 1B). Oral sorafenib therapy for 4 weeks was considered with the intention to close the ITS. During the interval of sorafenib administration, no side effects occurred. Re-evaluation by CT showed a potential closure of the ITS (Fig. 1C), which was confirmed by DSA (Fig. 1D). According to mRECIST, the patient was in partial response with a decrease in contrast-enhancing tumor mass from 4.0 to 2.1 cm. Technical, uncomplicated and safe TACE could be carried out with sufficient lipiodol saturation of the HCCs. Disease progression was observed 501 days [overall survival (OS) = 689] after initial sorafenib treatment.

**Fig. 2:**
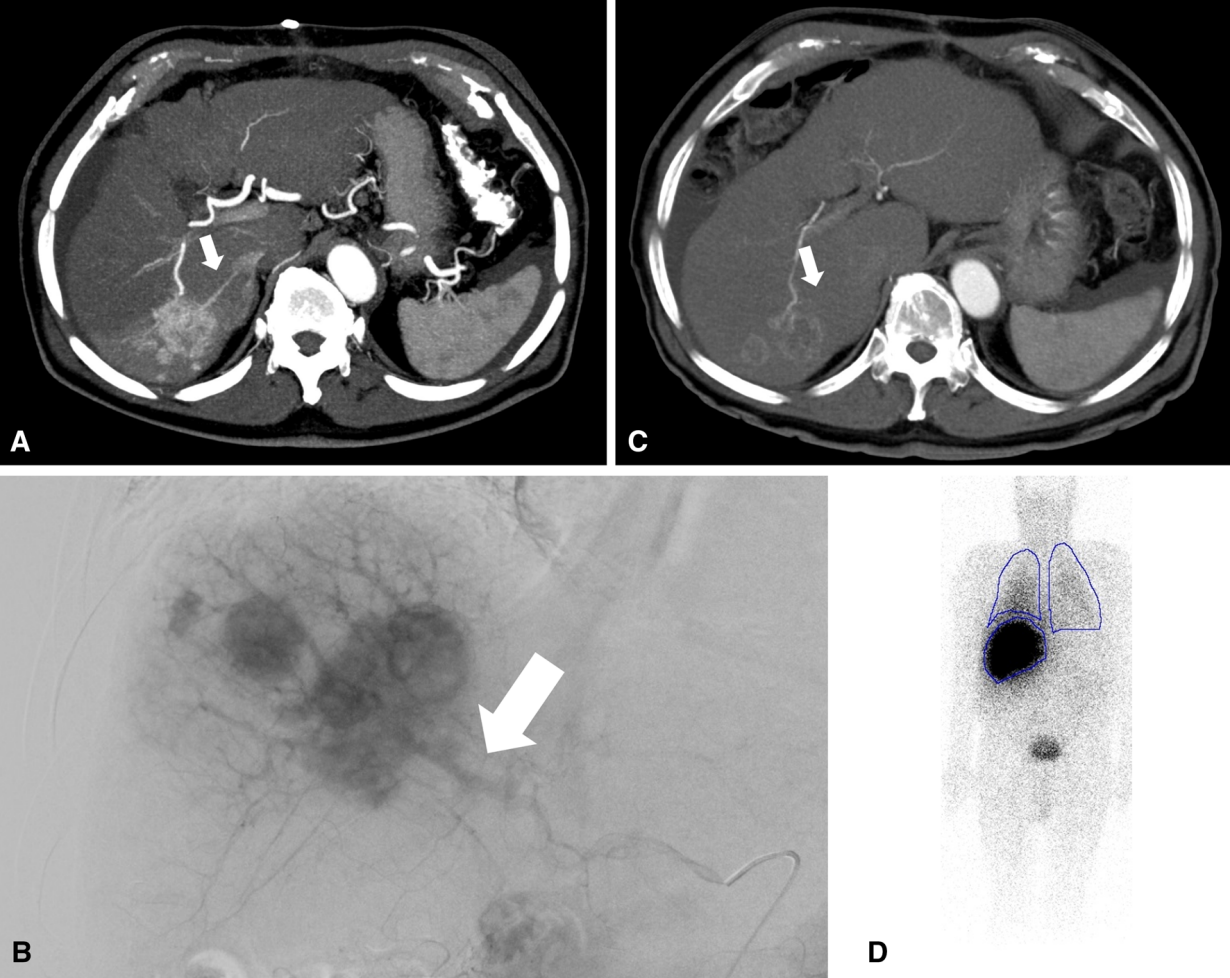
An 82-year-old patient with multifocal HCC in the right liver lobe due to hemochromatosis. **A** An arteriovenous fistula (white arrow) can be delineated based on the CT images. **B** DSA confirms the large arteriovenous fistula (white arrow) with high-flow conditions. **C** After treatment with sorafenib, a significant fistula cannot be visualized anymore. Although the portal vein is slightly contrasted, that might be caused by contrast through sinusoidal collaterals. In DSA no correlate for the previous fistula was found. Of note, even after short-term sorafenib therapy, HCC exhibits devascularization and central necrosis can be seen (white arrow). **D** MAA administration was carried out, and SPECT reveals a low HPS fraction with 9.9% (lung and liver are delineated in thin blue lines)

An 82-year-old man with multifocal HCC due to hemochromatosis underwent explorative laparotomy and was rated as inoperable due to multiple suspect lesions. Following the decision of MDT, preparation for TARE was completed with multiphase CT. With this, a significant ITS in segment VI with a single arteriovenous fistula was diagnosed (Fig. 2A, ESM 1). DSA showed a large high-flow fistula (Fig. 2B); therefore, the patient was excluded from intraarterial therapy, and MDT indicated oral sorafenib therapy. After 4 weeks of initial sorafenib administration, the patient showed distinct side effects with a sorafenib-associated hand–foot syndrome. The following CT showed complete closure of the ITS and a remarkable tumor shrinking (Fig. 2C, ESM 2). According to mRECIST, the patient showed a partial response with a decrease in arterial enhancing tumor mass from 1.9 to 0.5 cm (ESM 3). MAA administration and SPECT confirm the absence of a relevant HPS with 9.9% (Fig. 2D). Subsequently, TARE could be carried out with excellent tumor response. Progression-free survival was 488 days, and overall survival was 721 days.

**Fig. 3:**
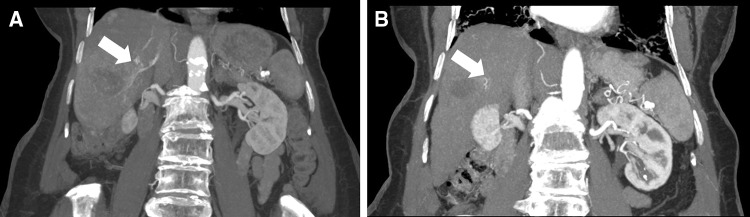
Coronary reconstructed maximum intensity projection of pre- and post-therapeutic CT of a 79-year-old patient with central HCC manifestations. **A** An arteriovenous fistula can be delineated based on the CT (white arrow). **B** After the treatment with sorafenib, the fistula cannot be visualized (white arrow)

A 79-year-old woman with multifocal HCC due to HCV was evaluated for TACE. The majority of HCCs were located in the right liver lobe with one large HCC in segment VI. Pre-interventional CT revealed a significant arteriovenous ITS (Fig. 3A). After four weeks of maximum oral sorafenib therapy (800 mg/day), MDT prolonged the treatment with a reduced dose (400 mg/day) due to cutaneous adverse effects, good tumor response but still patent ITS on CT. After 8 weeks of oral sorafenib therapy, CT revealed excellent tumor response with a closed ITS and a remarkable decrease in viable tumor mass from 2.2 cm to 0.4 according to mRECIST (Fig. 3B). Technical, uncomplicated and safe TACE could be performed subsequently with sufficient lipiodol saturation of the right hepatic HCCs (ESM 4). The patient showed a disease progression after 599 days and died after 630 days of initial sorafenib treatment.

## Discussion

Based on our clinical observation, closure of ITS with short-term oral sorafenib administration to make patients amenable to transarterial treatment is feasible, regardless of the presence of arterioportal or arteriovenous shunting.

To date, a solid body of scientific data indicates an interventional occlusion of a significant ITS. Murata et al. [5] reported an overall survival of 10.6 months (322 days) after embolization of ITS with coils, gelatin sponge, particles or a combination of these. However, interventional closure of ITS is technically demanding and may reduce the efficiency of following intraarterial treatment. In line with our clinical observation, Theysohn et al. [12] could report a significant HPS reduction (from 26.5 to 7.5% on average) after oral sorafenib before TARE. In contrast, Erxleben et al. [13] could not document a relevant difference of HPS in a comparison of patients treated with and without sorafenib before TARE (9.5% vs. 10.2%).

An increasing data basis indicates a combination of intraarterial approaches and sorafenib as a promising treatment option [14]. Although these findings are encouraging and suggest that this might be a feasible treatment option, the influence of sorafenib on the microvacation and therefore the outcome of TACE or TARE have not been completely analyzed to date. A basic mechanism of action for both TACE and TARE is the embolization of the tumor-feeding vessels. This leads to fairly selective hypoxic tissue damage within the tumor. In line with this, sorafenib is a multityrosine kinase inhibitor and therefore changes the tumor microvascularization, too. To which extent these effects are complementary or competitive, this cannot be derived by the current literature [15]. Concerning the good response to transarterial therapy that we did not only encounter in the presented cases, we strongly believe that this is a good therapy option also after sorafenib therapy.

Sorafenib as an orphan medication [16] has demonstrated to be effective for the treatment of patients with advanced-stage HCC with a reported overall survival of 10.7 months (325 days) [8]. By indicating sorafenib to patients with a significant ITS, we hypothesize that the anti-angiogenic effect of sorafenib causes a flow reduction in the shunt-supporting arteria system and leads to a closure of the ITS. In our clinical observation, all patients showed excellent disease control under intraarterial therapy, which exclusively was enabled through oral sorafenib administration. However, treatment of ITS remains difficult, and no sufficient therapy option exists in the rare case of significant ITS. Out of this, prospective studies should investigate the safety and efficiency of short-term oral sorafenib administration for patients with advanced-stage HCC and ITS to enable transarterial therapy.

## Conclusion

Based on our clinical observation and the limited clinical evidence, we suggest considering short-term oral sorafenib for patients with advanced-stage HCC and significant ITS as a therapeutic option with the intention to treat hereafter intraarterially.

## Electronic supplementary material

Below is the link to the electronic supplementary material.

Supplementary file1 (PPTX 1208 kb)

Supplementary file2 (PPTX 1539 kb)

Supplementary file3 (PPTX 336 kb)

Supplementary file4 (PPTX 107 kb)
