# Supplementary figures and images for: Short-Term Oral Sorafenib for Therapy of Intratumoral Shunts of Hepatocellular Carcinoma to Enable Intraarterial Treatment
Source: Cardiovasc Intervent Radiol. 2019 Jul 30;42(10):1494–9. doi: 10.1007/s00270-019-02294-7 (PMC6715807; doi:10.1007/s00270-019-02294-7)

## Slide 1
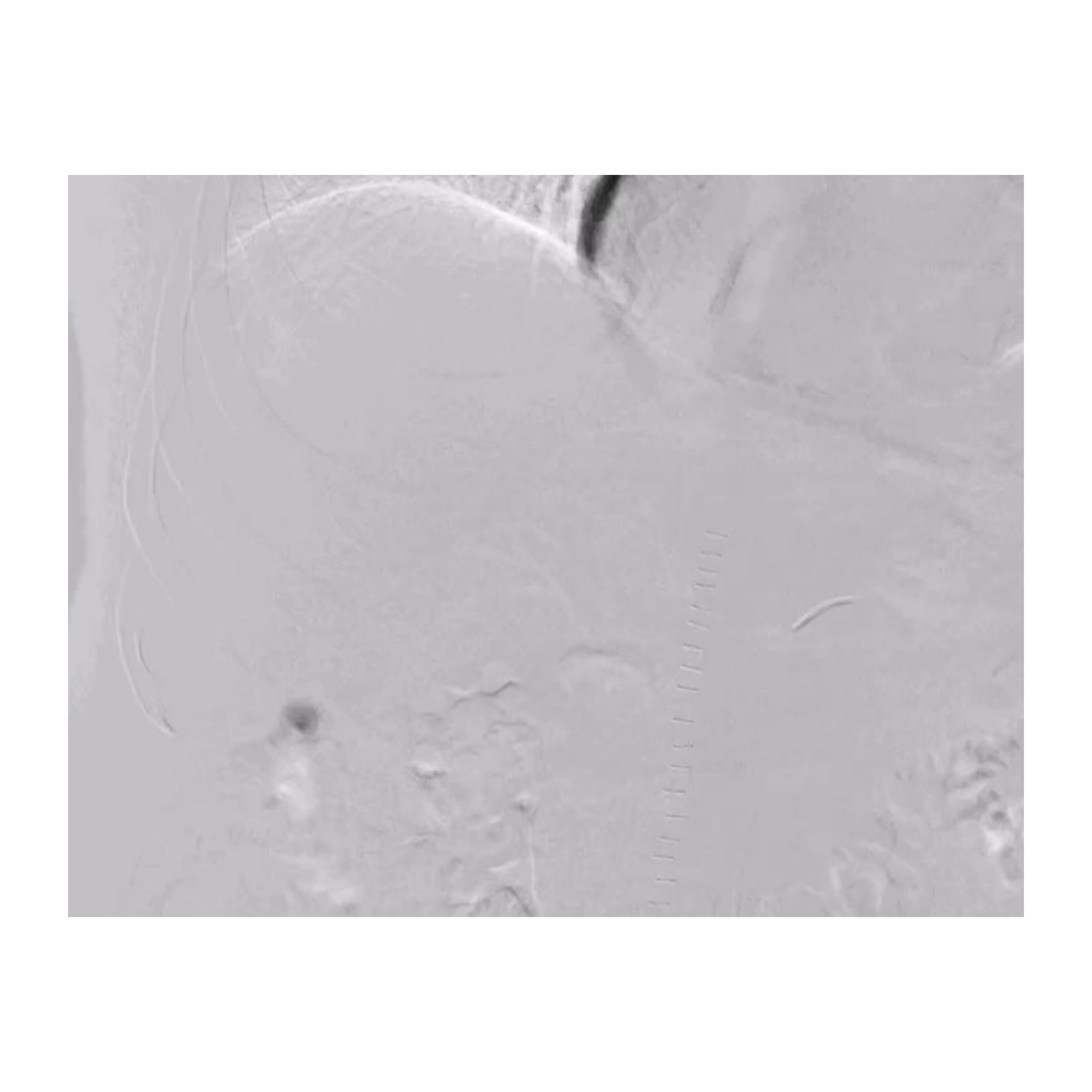

Supplement: Supplementary file 1 — Supplementary file1 (PPTX 1208 kb) [file 270_2019_2294_MOESM1_ESM.pptx]

## Slide 1
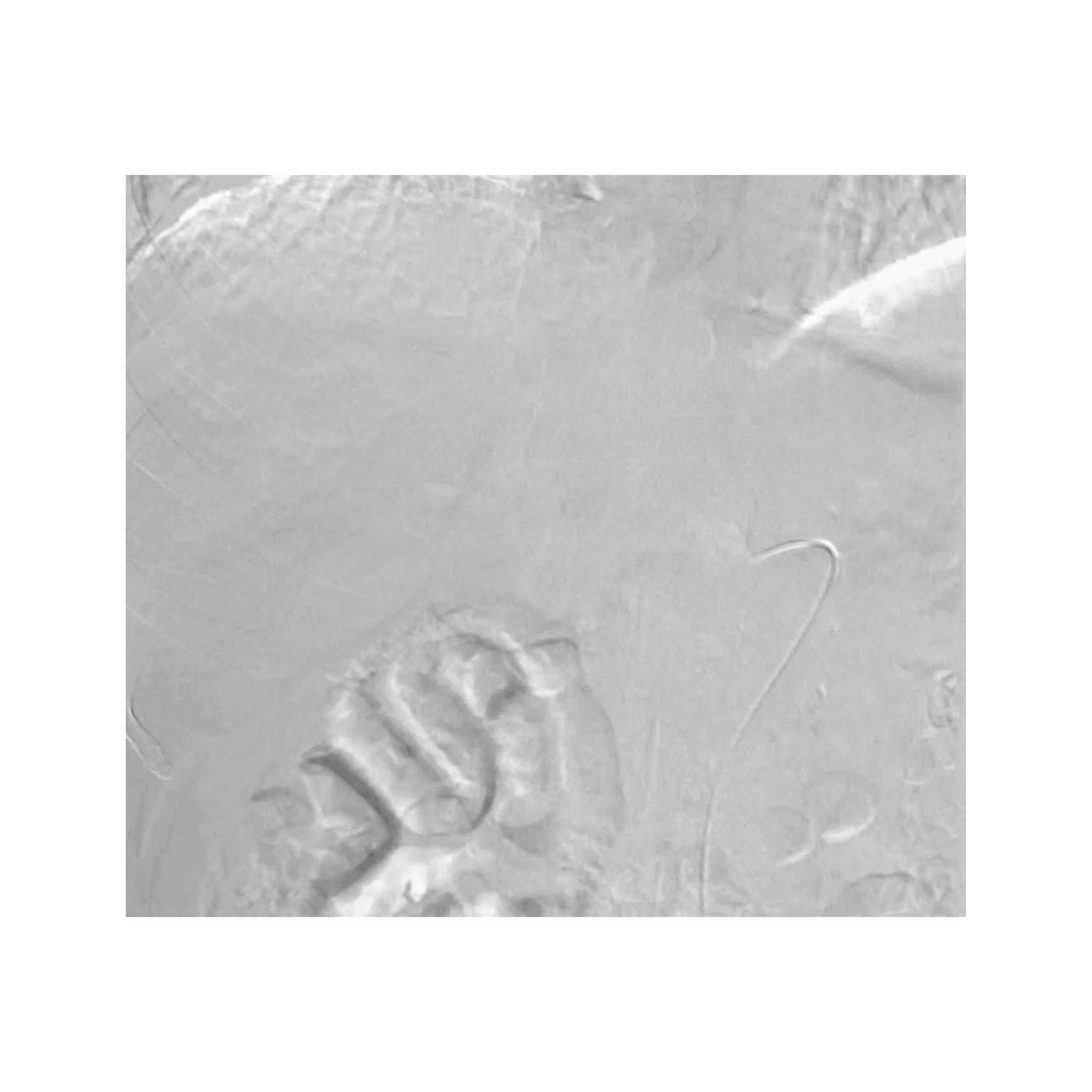

Supplement: Supplementary file 2 — Supplementary file2 (PPTX 1539 kb) [file 270_2019_2294_MOESM2_ESM.pptx]

## Slide 1
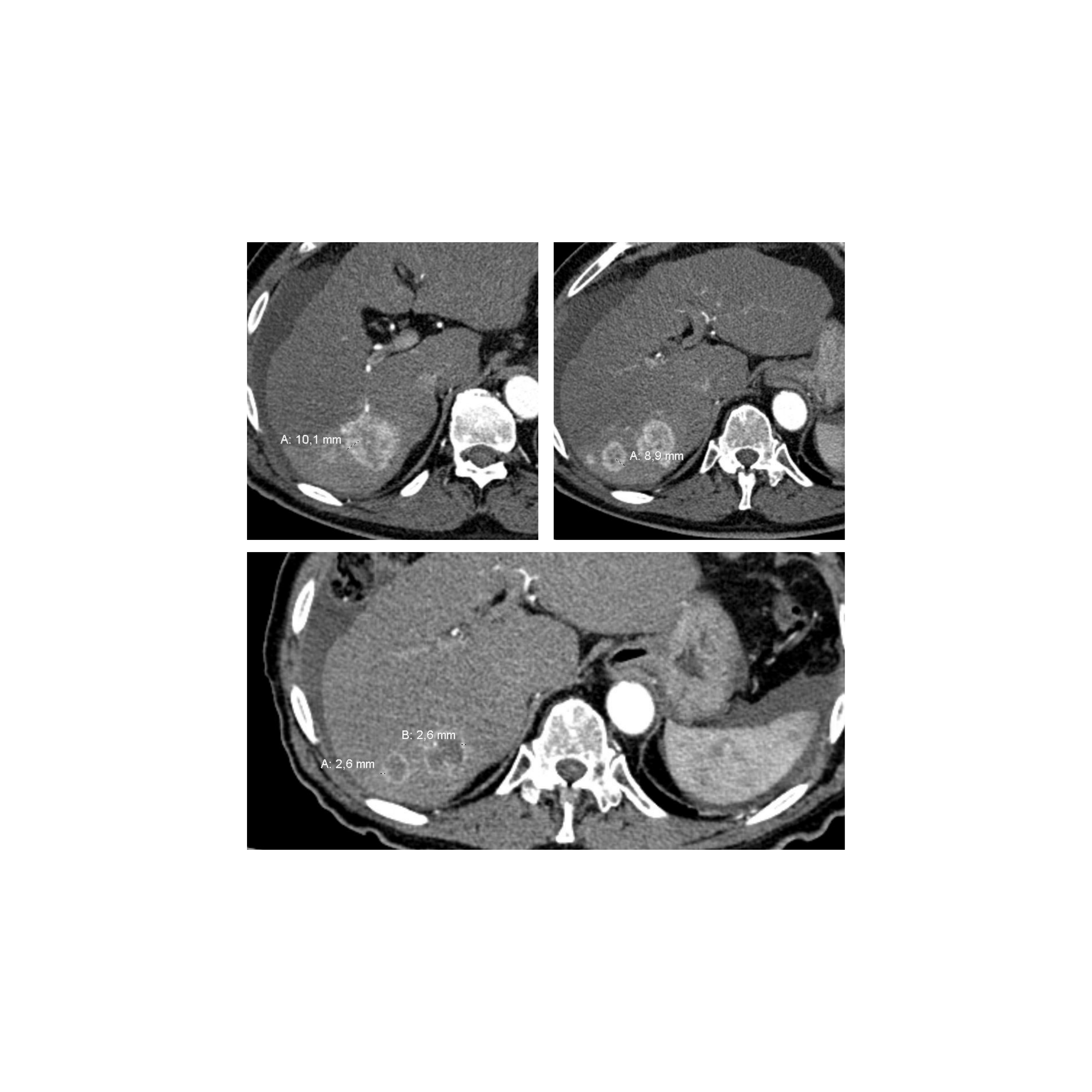

Supplement: Supplementary file 3 — Supplementary file3 (PPTX 336 kb) [file 270_2019_2294_MOESM3_ESM.pptx]

## Slide 1
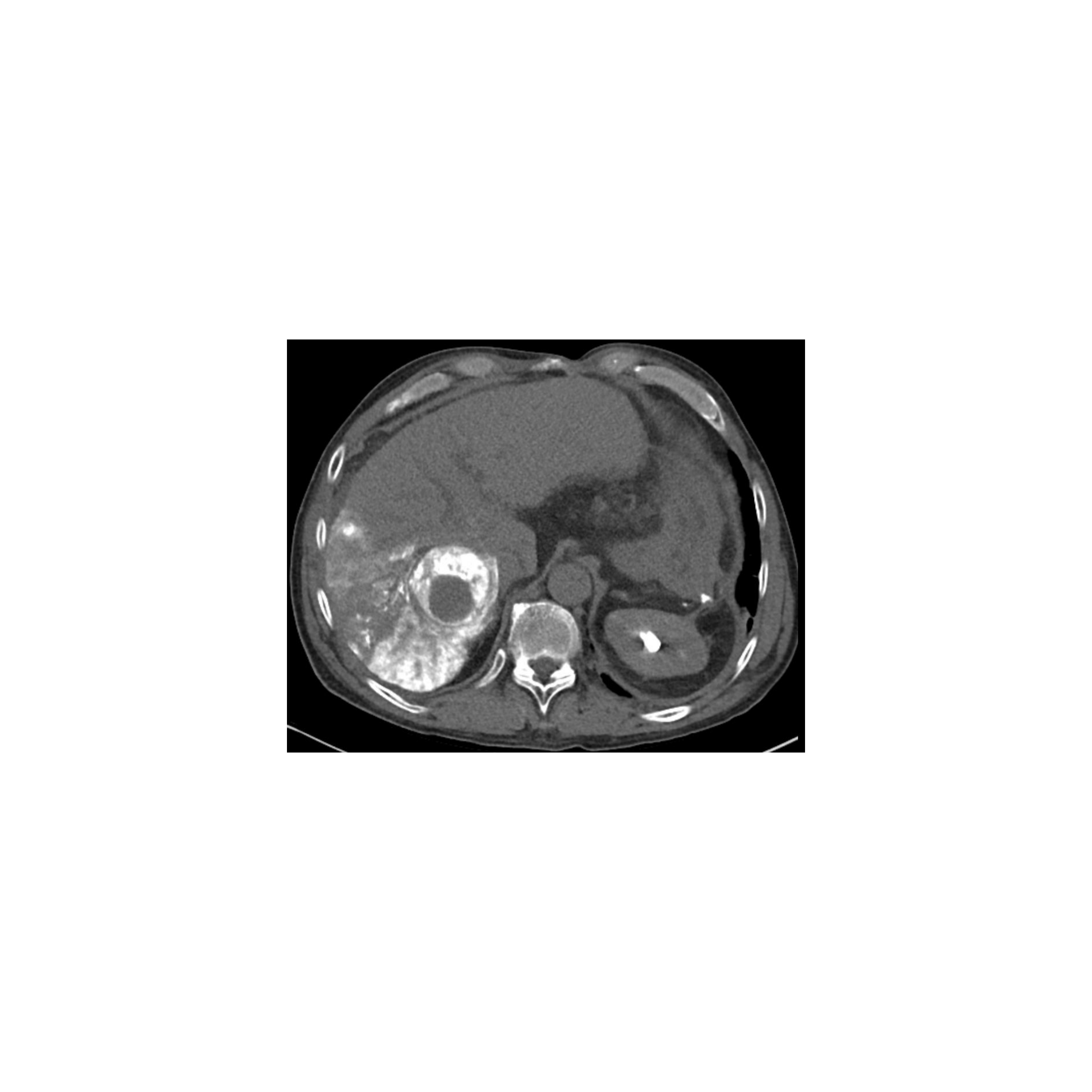

Supplement: Supplementary file 4 — Supplementary file4 (PPTX 107 kb) [file 270_2019_2294_MOESM4_ESM.pptx]
